# A rite of passage: a mixed methodology study about knowledge, perceptions and practices of menstrual hygiene management in rural Gambia

**DOI:** 10.1186/s12889-019-6599-2

**Published:** 2019-03-07

**Authors:** Vishna Shah, Helen M. Nabwera, Fatou Sosseh, Yamundao Jallow, Ebrima Comma, Omar Keita, Belen Torondel

**Affiliations:** 1 Environmental Health Group, Department of Infectious Diseases, London School of Hygiene and Tropical Medicine, Keppel Street, WC1E 7HT, London, UK; 2 Nutrition Theme, MRCG Keneba, Medical Research Council Unit, The Gambia, P.O.Box 273, Banjul, The Gambia; 3 Centre for Maternal and Newborn Health, Liverpool School of Tropical Medicine, Pembroke Place, Liverpool, L3 5QA UK; 4 Regional Education Directorate Four, Ministry of Basic and Secondary Education, Mansakonko, Lower River Region, The Gambia

**Keywords:** Menstruation, Knowledge, Preparedness, Taboo, Menstrual hygiene practices

## Abstract

**Background:**

Appropriate menstrual hygiene management (MHM) is impeded by taboos and secrecy surrounding menstruation. Unhygienic menstrual practices and unpreparedness for managing menstruation has been associated with adverse health and social outcomes among adolescent girls. In The Gambia, there is limited data on menstrual practices among girls and women in rural communities and the sources of information about menstruation for the adolescents. This study aimed to explore knowledge, preparedness and practices of menstruation and its management among adolescents, mothers and teachers in rural Gambia.

**Methods:**

A mixed methods study was conducted in the rural Kiang West district of The Gambia. Twenty focus group discussions and thirteen in-depth interviews were conducted among mothers, adolescents and teachers to explore their views on menstruation, cultural beliefs, sources and level of knowledge on menstruation and MHM practices. In addition, a survey was done among 331 school girls to assess their knowledge, practices and attitudes of menstruation and its management. Inductive content analysis was used to analyse the qualitative data, and descriptive analysis and chi-squared tests were used to analyse quantitative data.

**Results:**

All participants had different levels of knowledge about menstruation. Knowledge score was higher among post-menarche girls compare with pre-menarche girls (*p* = 0.0001). All groups expressed difficulties, embarrassment and shame in relation to discussing menstruation. Two thirds of the surveyed girls reported having learnt about menstruation before menarche, however at menarche most felt unprepared. Teachers were the main source of information, but when asking for advice most girls preferred to ask their mothers. Mothers reported facing difficulties in discussing menstruation with their children and felt that boys did not need to be taught about it, however boys were very curious to know about. Most girls used reusable cloth unless they are given free pads from school.

**Conclusion:**

Taboos, secrecy and embarrassment associated with discussing menstruation hinder adolescents from seeking advice from parents and teachers on appropriate MHM practices. Strategies to encourage positive social norms towards menstruation would help to promote more open discussions about it at the family, community and national level, which will support improvements in MHM in this and similar communities in low and middle income settings.

**Electronic supplementary material:**

The online version of this article (10.1186/s12889-019-6599-2) contains supplementary material, which is available to authorized users.

## Background

Menstruation is an important, physiological process that occurs in most girls during puberty [1–3]. Unhygienic methods and inappropriate environments for managing menstruation have been linked to adverse health and social outcomes. At an individual level, inadequate menstrual hygiene management (MHM) predisposes adolescent girls and women to urogenital infections, psychosocial stress and reduced opportunities for accessing school and work [1, 4–10]. All these consequences limit a woman’s ability to sustain herself and her family and ultimately impact a country’s economy [11]. The Gambian National Strategy for Sanitation and Hygiene 2011–2016 developed by the Ministry of Health and Social Welfare recognises the role of improved menstrual hygiene and sanitation at schools, in improving the academic performance and reducing school drop-out among adolescent girls [12].

To manage menstruation adequately and with dignity, women need access to information, hygienic absorbent materials, water, appropriate sanitary facilities that ensure privacy, positive social norms and adequate policies in place [2, 4, 13, 14]. Inadequate preparation before menarche leaves girls with negative feelings towards menstruation such as fear, confusion, and low self-esteem [1, 4, 14–17].

In many cultures, menstruation is considered a taboo [2, 18–23] and is clouded with silence and secrecy [4, 16, 18, 24].In The Gambia, menstruation is not only a taboo subject for public discussion, but also rarely spoken of in the private [25, 26]. This often leads to misconceptions and lack of preparedness among adolescent girls [1, 4–6, 21]. In addition, a review by Chandra-Mouli et al in 2017 suggests that across low- and middle-income countries (LMICs), the degree of guidance on menstruation from adults may contribute to the variations in basic MHM practices such as absorbent used, frequency of change and daily bathing practices [27].

Boys may also influence girls’ experiences of MHM [19]. They can support girls to manage menstruation effectively across different social domains including household, community and school [28]. However, little is known about knowledge and attitudes towards menstruation among boys in rural Gambia and the impact of involving them in MHM intervention strategies [19, 28].

Some of the menstruation related studies conducted in The Gambia have focussed on menstrual disorders or reproductive infections among adult women [25, 29]. However, there are still many gaps and unanswered questions about MHM among adolescents and women in The Gambia and in the West African region. This study therefore aimed to explore knowledge and perceptions of menstruation and its management among adolescents, mothers and teachers in these rural Gambia communities, and identify barriers to adequate MHM, that is key to developing effective MHM interventions.

## Methods

### Study site and population

The Gambia is the smallest country on mainland Africa [30], and is predominantly Muslim (95.7%) [31]. Almost 70% of the rural population lives in poverty i.e. a lack of food security and lack of access to good education, healthcare, electricity and safe water [32, 33].

**Fig. 1:**
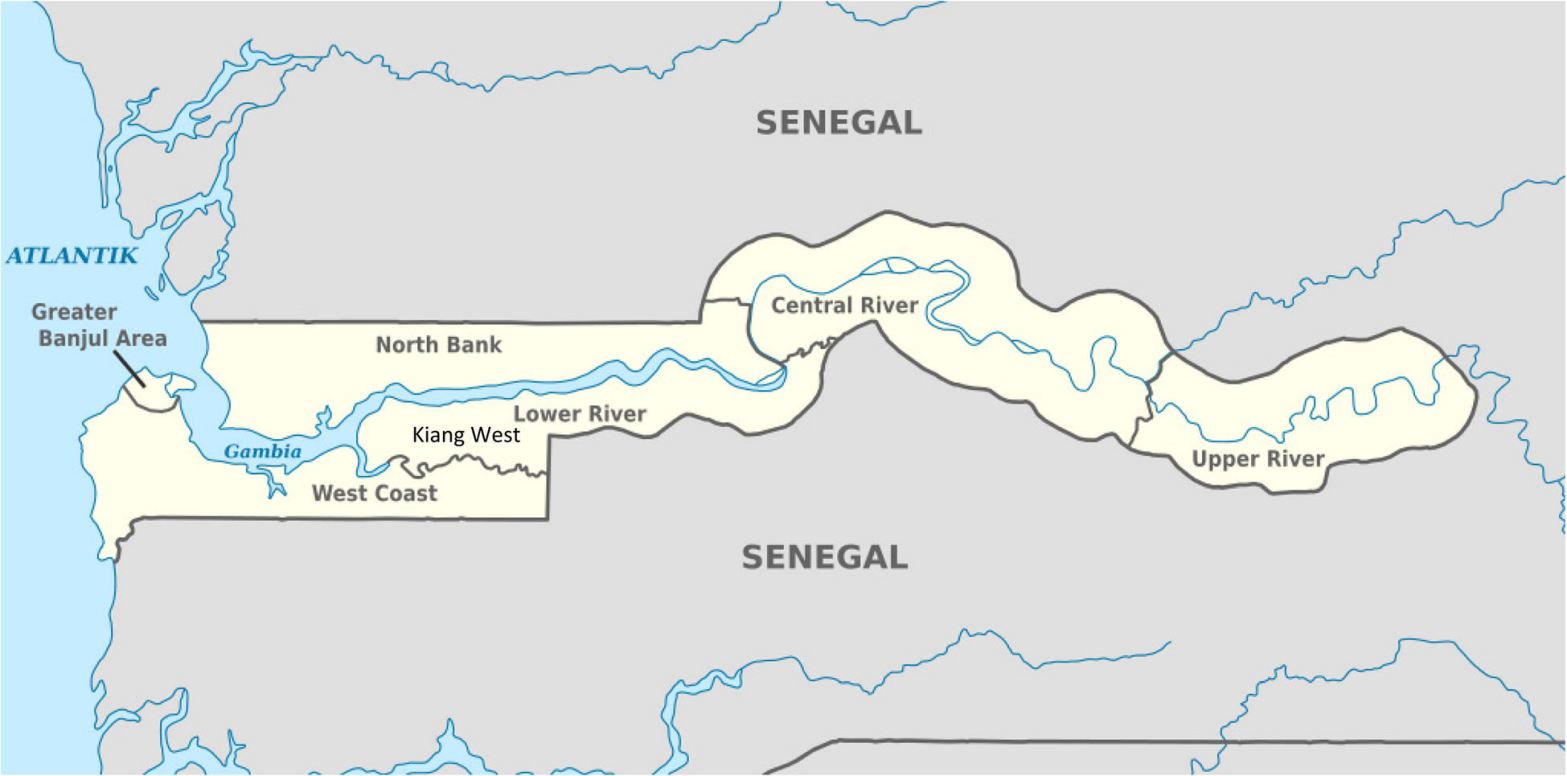
Map of the region where the study took place. Source: https://commons.wikimedia.org/wiki/File:Gambia,_administrative_divisions_-_de_-_monochrome.svg. Author: TUBS

The study was conducted in seven schools within four rural villages (Jali, Karantaba, Keneba and Manduar) of the Kiang West district of The Gambia (Fig. 1), where 99% of the population is Muslim [34]. The study was conducted between July 2015 to April 2016. The villages were within 10 miles of the Medical Research Council Unit The Gambia (MRCG) field station that provides free primary health care services and 24 h emergency care services [35]. This region has a population of over 15,000, 52% of who are women and 13% adolescent girls [34]. Schools in the region (and the rest of The Gambia) are broadly classified as English-based [6] or Arabic-based schools [1]. The English-based schools are free public schools, whereas Arabic schools are private and mainly focus on Quranic education [36].

Schools were selected through convenience sampling. The factors influencing selection included; recommendation from the senior education authority for that region, accessibility, existing working relationship between MRCG and the institutions and willingness of the headmaster to support the project. The schools then provided a list of all girls aged 11–21 years and boys aged 15–21 years.

### Study design and sampling

A mixed methods design was employed, combining qualitative and quantitative methods. Qualitative study was conducted first, where girls, boys, teachers and mothers were selected to participate in focus group discussions (FGDs) or in-depth interviews (IDIs). The qualitative study had two purposes; The main purpose was to enable us to understand the perceptions, cultural beliefs, knowledge and practices related to menstruation with this context. Secondly to help us to refine the questionnaire for the quantitative survey. In the development of the questionnaire, we initially drafted one using different existing survey tools on menstrual hygiene management used previously by the authors and other publicly available in the literature. We then used the qualitative data to refine the questions, to ensure that they were relevant and appropriate to the context and to identify and include questions related to any interesting issues that emerged in the qualitative study that were not included initially. The quantitative survey aimed to quantify information about practices, knowledge and attitudes about menstruation among adolescent girls. The survey also included questions on basic demographics, education and occupation of female and male heads, type of household constructions, availability of latrine facility, access to water and age of menarche.

#### Qualitative study

In order to select girls for FGDs, from the list provided by each school, two girls’ groups were created according to age: One was for girls between 11 and 15 years old (targeting pre-menarche girls) and the second one was for girls between 15 and 21 years old (targeting post-menarche girls). From each group, 10 girls were selected randomly using excel random selection. Girls were consented and asked their menarche status before the discussions, and then split into groups of pre- and post-menarche girls. For the boys FGDs, 10 boys (between 15 and 21 years old) were selected randomly using excel random selection from the list provided by each school. Our aim was to conduct at least one FGD for each group type in each school, to capture views and perceptions of the discussed topics from each school. For both the girls and boys FGDs, we invited 10 participants per group, expecting some absences on the day of the FGDs. We therefore managed to have between 6 and 8 participants per FGD. Two extra FGDs were conducted with post-menarche girls from two larger schools. In total twenty FGDs were conducted; nine FGD (64 participants) were conducted among post-menarche girls, five among pre-menarche girls (32 participants). For pre-menarche girls we only conducted five as two schools were secondary and did not have enough pre-menarche girls to create a group. Six FGDs (38 participants) were conducted among boys, we could not conduct a FGD in one school as only three boys showed up the day of the group discussion.

Thirteen IDIs were done; three with teachers, five with mothers, and five with boys. All the participants were purposively selected. Teachers who were interested in the study topic and wanted to share their experiences and opinions openly or worked closely with students on topics related to reproductive health were invited to participate in the study. Teachers suggested mothers that were vocal and active in the community and the school. Five of these mothers were then, approached to participate in the study. Two of the boys were selected because of their openness and engagement during the FGD while the other three were selected by recommendations from the teachers. Data saturation was the marker of sample adequacy [37].

#### Quantitative study

The sample size was calculated using data from a study conducted in Ethiopia [1] as there were no estimates available in The Gambia. The two scenarios for computing sample size were: using the prevalence of knowledge about menstruation at 92% and the prevalence of sanitary napkins use at 37.6%. 37.6% prevalence was taken, which gave us a larger sample size. Based on these assumptions, and using precision/absolute E of 5% and a type 1 error of 5%, a sample size of 361 was calculated. Seven out of nineteen potential schools ranging from primary level to senior secondary and Arabic schools within the Kiang West region were selected, using convenience sampling due to logistic reasons. At stage 2, 361 school girls (with an age range of 11–21 years) were selected from the list of female students provided by the teachers, using a stratified random sampling methods with proportionate allocation to the school size.

### Data collection

Data from students and teachers was collected in a private room on the school grounds, while that from mothers was collected in a private area within the community (space usually used for MRCG field visits). All data was collected in Mandinka (local language) by trained field assistants. The field assistants were trained on all aspects of the study procedures including setting up the room to facilitate FGDs and optimal communication strategies with the participants. Sessions with the girls, mothers and female teachers were conducted by a female field assistant, while those with the boys and male teachers were done by a male field assistant. As menstruation is a sensitive topic, same sex field assistants were selected to facilitate participation in the study.

#### Pilot testing

Qualitative and quantitative tools were first piloted using 3 Gambian nurses (2 men, 1 woman) and 4 Gambian field assistants (3 men, 1 woman) from MRC Keneba research centre. The purpose of this was to test translation and acceptability of questions and to support the training of field assistants. It was then piloted on 4 volunteers (1 adolescent boy, 3 adolescent girls) from the community to test, feasibility, acceptability and quality of questions and response answers available. Feedback from this pilot was used to amend the tools. The pilot also helped train data collectors, assess the best way to administer the FGDs and IDIs, and how to encourage all participants to participate equally.

#### Qualitative study

The interviews (Additional file 1) and FGDs (Additional file 2) were conducted by field assistants who were trained by the primary author in qualitative research methods. They explored views on menstruation, sources and level of knowledge on menstruation, parents and teacher’s views on how important it is for adolescents to know about menstruation, how menstruation is taught and approached, MHM practices, how menstruation has influenced a girl’s life, and boys’ reaction to menstruating girls.

The primary author was present for the FGDs and noted the non-verbal cues only, as she was not fluent in Mandinka. The IDI lasted about 25–40 min and the FGDs lasted about 30–70 min. FGD and IDI were recorded using a digital voice recorder.

#### Quantitative study

Since Mandinka is not a written language and most participants are not fluent in English, the questionnaire could not be self-completed. The survey was conducted individually. The trained field assistants read out the questions and noted the participant’s answers. Questionnaires captured information about socio-demographic factors, age of menarche, knowledge and attitudes about menstruation, menstrual hygiene practices (such as type of menstrual absorbent used, change frequency, washing and drying practices of reusable material), and factors affecting hygienic management (Additional file 3).

Level of knowledge score was calculated using the 5 knowledge specific questions described in Table 4. Correct answers were assigned ‘1’ and all false or don’t know answers were assigned a ‘0’. Thus, the highest possible score attainable, with all correct answers is 5. Scores from 3 to 5 were judged to have good knowledge and 0–2 as poor knowledge.

Completeness and consistency was reviewed at the end of each data collection day by the primary author.

### Data analysis

#### Qualitative

Data from the IDI and FGDs was simultaneously translated and transcribed into English by the field assistants who conducted the interviews. For quality purposes two interviews were sent to an independent second transcriber to ensure accuracy of transcripts. Any inaccuracies found were discussed by all the transcribers and the primary author, in order to understand the root of the differences.

Data was managed using Ms. Word and Ms. Excel. Inductive content analysis was conducted [38]. The primary author and the second author independently read the transcribed data carefully and segmented the data. Meaningful segments were assigned a code by each researcher and the codes were then discussed and compared. The first author analysed all the transcripts. Six random selected transcripts were assigned to the second author for analysis to test the inter-rater reliability regarding the codes and themes emerging from the transcripts.

#### Quantitative

Data was double-entered using SQL and analysed using Stata14 [39]. Descriptive statistics were used to summarize socioeconomic data, information regarding knowledge of menstruation and its management, source of knowledge, types of absorbent used and experiences while menstruating. Comparisons of knowledge between menstruating girls and non-menstruating girls was done using chi-squared statistics.

## Results

### Study participants characteristics

Four hundred seventy-eight participants from the four rural villages in Kiang West district were recruited into the study from July 2015 to April 2016. All the participants were Muslims residing in a rural setting. Table 1 shows the number of participants and their mean age participating in each component of the study.

**Table 1:** Overview of type of methods used in the study, participants number and characteristics in Kiang West District

Tools	Participants	Number of groups	Number of participants	Mean age of participants [years±SD]	Age range of participants (years)
FGDs	Post menarche girls	9	64	15.7 ± 1.4	15–21
	Pre menarche-girls	5	32	13.2 ± 1.0	11–15
	Boys	6	38	16.7 ± 1.1	15–21
IDIs	Female Teacher	1	1	–	30
	Male Teachers	2	2	–	37–45
	Mothers	5	5	–	27–49
	Boys	5	5	–	15–21
Questionnaires	Girls	–	331	15.3 ± 2.7	11–21
	Total	–	478	–	11–49

**Fig. 2:**
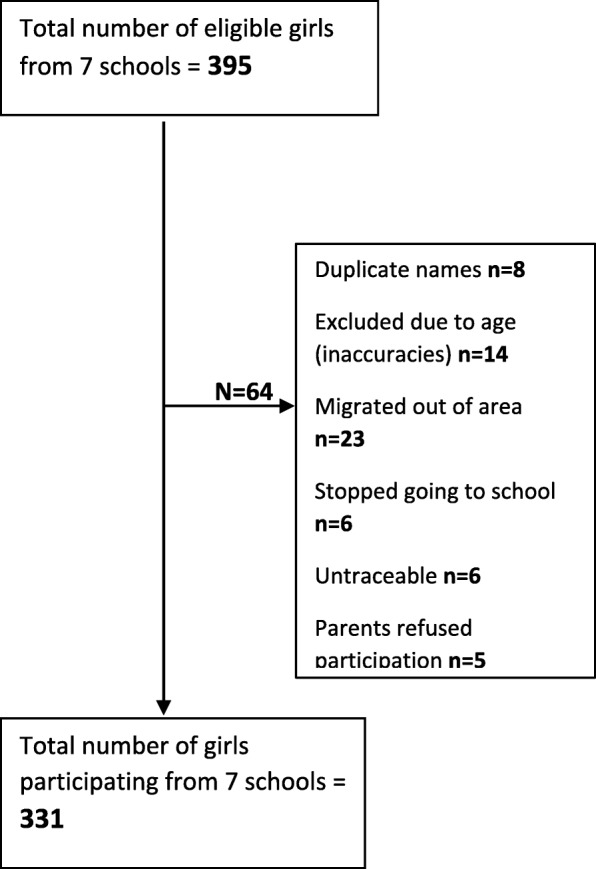
Flowchart of recruitment of girls’ participants in the cross-sectional survey from seven schools of Kiang West District

331 (91.7% of expected sample size) girls participated in the cross-sectional survey (Fig. 2).

Mean age of reported menarche among girls participating in the survey was 13.8 (± 1.4) years. There were mothers (14%) that had received no formal “English” education (Table 2). Most mothers and fathers had received only Arabic/Quranic education, 63 and 60% respectively. Two-thirds of girls reported that their households main source of income was through farming. 85% reported having access to piped water but only 9% had access to water in their compound. 87% had access to pit latrines with slabs, which are the most common types of toilets found in these communities.

**Table 2:** Sociodemographic characteristics of adolescent girls participating in the cross-sectional survey in the Kiang West district

Characteristics of respondents	Number	Percentage
Age group (years) (*n* = 331)		
11–13	106	32.0
14–16	138	41.7
17–20	87	26.3
Religion (*n* = 331)		
Muslim	331	100
Education level HH head, (*n* = 331)		
None	13	3.9
Primary school not completed	6	1.8
Primary school completed	4	1.2
Secondary school not completed	12	3.6
Secondary school completed	27	8.2
Arabic	197	59.5
Girls did not know	67	20.2
Education level caregiver, (*n* = 331)		
None	47	14.2
Primary school not completed	5	1.5
Primary school completed	4	1.2
Secondary school not completed	12	3.6
Secondary school completed	6	1.8
Arabic	207	62.5
Girls did not know	50	15.1
Material of walls of the house (*n* = 330)		
Mud	202	61.2
Cement	118	35.8
Other	10	3.0
Material of floors of the house (*n* = 330)		
Mud	77	23.3
Cement	237	71.8
Tile	15	4.5
other	1	0.3
Main source of income (*n* = 330)		
Farming	217	65.8
Salaried labour	52	15.8
Business	59	17.9
Other	2	0.6
Guaranteed income every month (*n* = 330)	94	28.5
Water Source, (*n* = 330)		
Piped water	280	84.8
Borehole (hand pump)	47	14.2
Protected well	1	0.3
Unprotected well	2	0.6
Location of water source, (*n* = 330)		
In own dwelling	30	9.1
Outside compound (community)	300	90.9
Toilet facilities (*n* = 330)		
Flush toilet	4	1.2
Pour flush toilet	2	0.6
Pit latrine with slab	286	86.7
Pit latrine without slab	37	11.2
Bush	1	0.3

### Qualitative and quantitative findings

Results from the qualitative study complemented with the information obtained in the survey are presented using 5 main topics; *1. Knowledge of menstruation; 2. Preparedness and support from family and teachers; 3. Challenging attitudes and emotions associated with menstruation; 4. Hygiene practices and menstrual absorbents while menstruating and 5. Cultural beliefs and restriction* (Table 3).

**Table 3:** Content analysis framework

Theme:	Girls experience while menstruating can be affected by personal [knowledge], social [support] and environmental factors [materials and physical environment].				
Category	1.Knowledge of menstruation	2.Preparedness and support from family and teachers	3.Challenging attitudes and emotions associated with menstruation	4.Hygiene practices and menstrual absorbents while menstruating	5.Cultural beliefs and restriction
Codes:	Prior knowledge	Timing of learning about menstruation	Secrecy	Washing and drying of absorbent material	Impurity
	Gaps in knowledge	Source of information	Embarrassment	Preference of absorbent material	Restrictions
	Misperceptions	Support with absorbents	Fear	Availability of sanitary pads	
	Illness/disease		Fertility	Knowledge gap on how to use sanitary pads	
				Malpractices	

Adapted from [1]

### Knowledge of menstruation

#### Qualitative findings

All participants had knowledge about menstruation, however levels of knowledge were different among participants, and there were misconceptions and gaps, especially in their physiology knowledge.

*“It is sperm” [Boys-FGD4&6- and Pre-menarche girls-FGD5]*

*“Special water comes out of the girl” [Boys-FGD4]*

*“The blood comes from the middle of your head and then comes down to your private parts” [Pre-menarche girls-FGD3].*

The most comprehensive explanation of menstruation came from a FGD with boys.

*“They have eggs inside, if that egg breaks…blood will come out of their body…If the man has sexual intercourse with the woman when she has an egg inside, the egg may not break…and she will not menstruate” [Boys-FGD2].*

Majority of participants, including mothers, did not know why women menstruate, while others linked menstruation to a religious folklore. None of the accounts linked menstruation to changes in hormonal levels.

*“If they have sexual intercourse with a man, you will see blood will come out” [Boys-FGD1]*

*“In the heaven, God asked Hawa, not to eat a fruit, and she happened to eat and swallow it, this is why women used to see menses… but Adam did not swallow it, that is why it got stuck in his throat and men have an Adams apple.” [Post-menarche girls-FGD7]*

*“It is a matter of must” [IDI mother2]*

The concept that “menstruation is a disease” was seen during the FGDs with girls as well as interviews with mothers and teachers.

*“When it attacks her, she must go home to look after herself” [IDI Teacher1]*

*“…That thing must come out of her” [IDI Mother 3]*

#### Quantitative findings

Some girls [9%] reported not having heard anything about menstruation, of which 30% had reached menarche. Quantitative data also confirmed the findings that a high proportion of girls (a third) thought menstruation was a disease (Table 4), while a further 23% were not sure whether it was a disease or not. Less than half of the participants knew that menstrual blood comes from the womb. A fifth of the participants thought that very old women and pregnant women could menstruate, whilst around a third of participants was not sure. Post-menarche girls had a higher knowledge score than pre-menarche girls (*p* = 0.001) (Table 4).

**Table 4:** Adolescent school girl’s knowledge about menstruation from cross-sectional study (All girls N = 331, Pre-menarche girls *N* = 128, Post-menarche girls, *N* = 203)

	All n [%]	Pre-menarche n [%]	Post-menarche n [%]	Test of significance*
Questions				
Very old women menstruate	62 [18.7%]	39[30.5%]	23[11.3%]	*p* = 0.001
Menstruation is a disease	103 [31.1%]	45 [35.2%]	58 [28.6%]	*p* = 0.03
Pregnant women menstruate	63[19.0%]	49 [38.3%]	14 [6.9%]	*p* = 0.001
Menstrual blood comes from the stomach	25 [7.6%]	13 [10.2%]	12 [5.9%]	*p* = 0.19
Menstrual blood comes from the womb	155 [46.8%]	62 [48.4%]	93 [45.8%]	*p* = 0.7
Knowledge score				
Poor [0–2]	163 [49.2%]	94 [73.4%]	69 [34.0%]	*p* = 0.001
Good [3–5]	168 [50.8%]	34 [26.6%]	134 [66.0%]	

*chi square between girls that already have their period compared with girls without it

### Preparedness and support from family and teachers

#### Qualitative findings

During the FGDs, the girls reported that they learnt about menstruation from their teachers first and also from mothers or sisters.

Mothers felt that it was the duty of the teachers to tell their daughters about menstruation.

*“They will learn it in school…the teachers know more than we…they also have books” [IDI mothers2]* The teachers acknowledged the importance of teaching girls about menstruation in school, but there is also felt that there was a need for parents to take a more active role in teaching their daughters about these issues.

*“Parents need to play a bigger role in talking about these matters…mothers need to be told not to be ashamed to talk to their daughters about menstruation.” [IDI- Male teacher1]*

Mothers reported that they would want their daughters to know about menstruation before they reach that stage, so that they are prepared and can take care of themselves when they away from home. However, the same mothers, also said they will tell their daughters about menstruation, when they see that they have started menstruating.

*“If I notice that they have seen it, I will explain it to them that the cloth people, how to use it” [IDI mother4].*

Only few girls mentioned that they would talk to their fathers about menstruation, while the rest said that they would never do that.

*“Aii (Ahhhh) my father… that I can never do…men are not meant to know” [Post-menarche girl IDI2].*

Information adolescents received in relation to menstruation was mainly related to religious Islamic requirements: abstaining for sexual intercourse to prevent pregnancies, religious restrictions such as not praying or fasting, the particular way to wash after they finish menstruating. In addition, they reported that they were usually shown what cloth to use to absorb the blood and informed that there is a lot of pain associated with menstruation. This was confirmed by the mothers as well, when they were asked what they would tell their children about menstruation.

#### Quantitative findings

A third of the surveyed girls reported they learnt about menstruation after they had their menstrual period. Girls reported teachers (78%) as the most frequent source of information about issues regarding menstruation, followed by mothers (28%), peers (20%) and sister (13%). These results support the findings observed with the qualitative data.

All post menarche girls knew of at least one absorbent material, while 11% of pre-menarche girls knew of none of the absorbent materials available. Pre-menarche girls were more aware of reusable cloth (rags) (76%), than disposable pads (59%). The same trend was seen in post menarche girls, 97% of post menarche girls knew about reusable cloth and 87% knew about disposable pads. Only a few mothers knew about disposable pads and how to use them.

Despite having heard about menstruation before menarche, 33% of the girls were not well prepared for it. They did not know what it was when they first experienced it. This confirms information from the FGDs and IDIs that mother have difficulties talking to their daughters about it, and if they talk about it, they do not share the practical aspects, so girls do not know what they will experience when they start menstruating. They only know the restrictions. Only 3% of girls reported they were expecting their menses.

### Challenging attitudes and emotions associated with menstruation

#### Qualitative findings

Embarrassment was the prevailing emotion reported in relation to menstruation, largely due to the need of secrecy and silence surrounding menstruation. Most girls in both the pre-menarche and post-menarche groups would try hide their faces in their scarfs or hands, fidgeted or laugh nervously, when menstruation was brought up. This was even seen with some mothers.

*“You feel panicked and you feel ashamed if you hear someone saying it, especially a man, you don’t like it.” [Post-menarche girls-FGD7]*

A recurring theme throughout the discussions was the importance of maintaining secrecy around menstruation. This was also emphasised by the teachers and mothers. Some girls thought it was too private to share even with their own mothers.

*“I didn’t tell anyone, I didn’t feel I could tell anyone, one of my friends found out so I told her, but I never told my parents.” [Post-menarche girls FGD 5]*

*“The moment you hear someone say period, even a woman, you feel ashamed, because it has to be something secret” [Post-menarche girls-FGD7]*

Mothers felt it was not important to discuss issues related to menstruation with boys, because they do not experience it. However, most boys were curious to know more about it and were more engaging in the discussions than the girls. This curiosity could also be seen by the questions they asked the facilitators.

*“They should not know…it is not important…It is not their way. It is a way for women.” [IDI mother1]*

*“Parents usually don’t talk about menstruation, but if they do, boys are not present.” [Boys-FGD1]*

*“Boys should know about menstruation…if you know, you can teach your wife how to be clean and pure again” [Boys-FGD4].*

Teachers felt embarrassed to talk about menstruation with students. Some felt that a male teacher should not be talking to girls about it because men do not know much about it, and both the teacher and the students would be uncomfortable and embarrassed. Therefore, it was the responsibility of the female teacher if she felt comfortable. However not all schools had a female teacher, such schools experienced difficulties when it came to discussing menstruation, either the topic was rushed over, or skipped all together.

*“It is difficult for teachers to teach it, even myself at first I used to feel ashamed to teach it, and then I feel I am a woman and I should teach them, because not knowing is dangerous…but even the girls, they feel ashamed when we teach it, and they are ashamed to ask teachers for advice.” [IDI-Female teacher1]*

Fear was another dominant theme seen in most discussions. Fear was mainly in relation to others finding out their menstrual status due to staining or because of not praying (Islam says girls are not allowed to pray when then are menstruating). Discussions also echoed concerns over fear of unknown, pregnancy and bullying.

*“If a girl is sitting with boys, she is constantly worried about what will happen if she starts bleeding or stains her clothes” [Boys-FGD2]*

*“Some girls pray even while menstruating just so that they can avoid others teasing them and knowing they are menstruating” [IDI-Female teacher1]*

Some girls reported being scared when they saw blood from the first time.

*“When I saw blood for the first time from down there, I was really scared…even though I heard it in school, I didn’t know what it was when it came.” [*Post-menarche girls-FGD4]

“I was worried, I thought there was a wound inside me” [Post-menarche girls-FGD2]

All the groups interviewed stated that menstruation is important for health and indicator of woman’s fertility.

*“Me, I remember them saying, if a woman does not see her menses, she will not have a child…the blood must come out of you otherwise the sickness will stay inside and that can cause problems to the girl” [Post-menarche girls-FGD3]*

However not all girls perceived menstruation as an advantage, because when they start menstruating they are less likely to play with their friends and there is a high chance they will have to get married soon.

*“If they see their menses for the first time they become worried, they fell they will get married soon” [Pre-menarche girls-FGD2]*

#### Quantitative findings

**Fig. 3:**
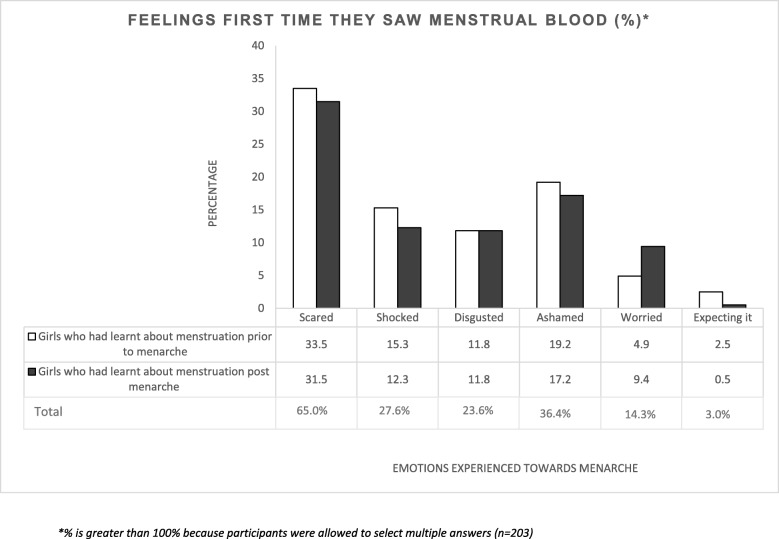
Summary of participant’s feelings first time they reach menarche

High levels of feelings of fear related to experiencing menstruation reported in the qualitative interviews were also found in the quantitative data: 65% of the participants said they felt scared the first time they saw menstrual blood, of which 51.5% had learnt about menstruation before menarche (Fig. 3).

### Hygiene practices and menstrual absorbents while menstruating

#### Qualitative findings

In this community, reusable cloth was the most frequent type of material used to absorb menstrual blood. Some girls and mothers reported preferring to use reusable pads because they can be used multiple times and they were easier to use. Mothers and even some girls reported not knowing how to use the disposable pads. One of the mothers had an overall idea on how to use the disposable pads but had some inaccurate practices.

*“If you bleed too much, you stick one pad first on the pant, then you put another one on top…you can put as many as you need.” [IDI Mother3]*

Majority of the girls who used disposable pads received them for free from school, who got their supplies from the government. The schools got a limited supply, so girls were often not given enough pads, as a result many resorted to changing their pads less frequently, to ensure the supply lasted for longer. Once the supply of disposable pads from school run out the girls tended to use cloth for the remainder of the menstrual period. Girls and mothers reported in the FGDs that disposable sanitary pads were too expensive or not available for purchase in their village shops or they were too embarrassed to buy it from the shop, especially if the shop keeper was a man.

*“Most girls get pads from their school, but this school doesn’t get a supply of pads so most girls use rags…pads are not sold in the village” [Post-menarche girls-FGD7]*

*“Pads are too expensive to buy, so if there is no supply in school we have to use rags.” [Post-menarche girls-FGD4]*

Secrecy around menstruation was seen in the way girls and women dry their reusable absorbent material.

*“Some people dry it in the house…under the mattress, or if you don’t do that, you must put it in a nylon bag and let it dry there” [Post-menarche girls-FGD3]*

*“You dry it in the bathroom, but it should be hidden” [Post-menarche girls-FGD9]*

#### Quantitative findings

The most frequent type of material reported in the questionnaire was solely reusable cloth (42%), which is consistent with the qualitative findings. This is followed by solely disposable pads (35%) or combination of both (23%) (Table 5). Majority (72%) of girls dried their material in the bathroom, at home, which are only used by women. Few (6%) girls even resorted to hiding the wet material under their mattresses. Less than 1% of girls in the study reported drying the material in the sun. Most girls washed their reusable pads with water and soap/detergent. Fifty-nine percent of post menarche girls reported changing the pads three times or more in a day (Table 5). Almost all girls (96%) reported that they change their menstrual absorbent in their bathrooms. Normally houses in these communities have a designated bathroom for women of the house and another for men in the house. The bathroom is completely separated to the house latrine.

**Table 5:** Menstrual hygiene management practices among adolescent girls from the cross-sectional study

	N (%)
Material used (*n* = 203)	
Disposable pads only	70 (34.5%)
Cloth only	86 (42.4%)
Both disposable pads and cloth	46 (22.7%)
Panties/Knicker only	1 (0.5%)
Change frequency (*n* = 203)*	
Once a day	10 (4.9%)
Twice a day	73 (36.0%)
Three times or more	120 (59.1%)
Washing material at home (*n* = 134)*	
Water only	8 (6.0%)
Water and soap/detergent	126 (94.0%)
Drying the material (*n* = 134)*	
In the sun or open space	1 (0.7%)
In the bathroom	96 (71.6%)
Inside the house	29 (21.6%)
Under the mattress	8 (6.0%)
Not buying absorbent material from the shop (*n* = 50)	
Too expensive	22 (44%)
Not available for purchase	21 (42%)
Too embarrassed to buy it from the shop	7 (14%)

*(in one of your more heavy days of bleeding)

Of those that used cloth only, 33% of them went to schools that provided free disposable pads. These girls tended to not get the disposable pad supply from school because they were too embarrassed or shy to ask for them, they did not know how to use disposable pads, or preferred cloth.

### Cultural beliefs and restrictions

#### Qualitative findings

Menstruation was considered to be a period of time when a girl/woman was impure and unclean, which is why a girl must adhere to the religious restrictions while she is menstruating. She must purify herself before she can return to these practices.

*“The Ustas (Islamic scholar who teaches about Islam) teach the students how to clean and purify themselves after menses…they are told when they should wash this way. Until you purify yourself you cannot be part of the society.” [IDI- Male techer2]*

Religious restrictions for menstruating women such as praying, touching the Quran, entering the mosque, and fasting during Ramadan were emphasised in all discussions. These religious and social restrictions prevent girls/women from undertaking their daily activities including attending school.

*“She should not sit in crowed places” [Boys-FGD4]*

*“She shouldn’t cook…when menstruating their hygiene is not good, which is why they are told not to cook” [Post-menarche girls-FGD3]*

*“If a girl plucks a lemon from the tree while menstruating, the tree will die” [Pre-menarche girls-FGD1]*

Some participants suggested that some methods of storing or disposing absorbent material can lead to infertility. Most post-menarche girls and mothers were strongly against burning used absorbent material.

*“You must throw the rag or pad in the latrine, they say if you burn it you will never have a child” [Post-menarche girls-FGD6]*

*“If your co-wife (*in a polygamous marriage, another wife of a woman's husband) *sees it [rag] she can do juju [curse] on you…so that you will not bear children” [Pre-menarche girls-FGD5]*

## Discussion

Our qualitative and quantitative findings show that menstruation is a taboo that is rarely discussed in these rural communities in The Gambia, affecting knowledge, perceptions and menstrual practices among women and adolescents. Adolescents and mothers had different levels of knowledge about menstruation, and many misconceptions were found. The main knowledge sources girls had for menstruation were teachers, mothers, sisters and peers. Our quantitative findings showed that girls who had started menstruating had better levels of knowledge compared with the ones that did not have their periods yet. However, learning about menstruation at the pre or post menarche stage did not have a significant difference on feelings experienced when seeing menstrual blood for the first time. Women in this community mainly used cloth as menstrual absorbents and most girls used cloth unless they were given free pads from school. Qualitative findings showed that cultural and religion beliefs were associated with restriction to normal daily activities when menstruating as well as with some of the menstrual hygiene practices (such as type of absorbent use, drying, storing and disposing practices).

Although teachers acknowledged the importance of teaching girls about menstruation in school, some teachers felt embarrassed and ashamed when talking about menstruation with students. They also felt it was inappropriate for a male teacher to talk to girls about menstruation. In addition, teachers did not feel fully competent to teach adolescent girls about menstruation and its management as they had not been formally trained to do this. UNESCO policies emphasise that both male and female teachers should be well informed and confident on issues related to menstruation and menstrual hygiene practices, which in turn will allow them to support female students and create an encouraging environment in school [40–42]. This is especially important in LMICs where there are very few female teachers [42]. Mothers also found it difficult to talk to their children about issues related to menstruation but felt that girls should know about menstruation before they reach menarche. Some of the reasons they gave were that they lacked confidence in talking to their daughters about menstruation because they felt they did not have sufficient information about the topic, this is also seen in other contexts as well, such as Kenya and Ethiopia [44–46]. Lack of communication between parents and children, as well as schools covering the issues with insufficient depth are also seen in Ethiopia [1], Nigeria [16], India [23] and Kenya [43], where menstruation is still considered a taboo. This could partly explain why girls are not well prepared for menarche or have negative feelings towards menstruation.

Mothers felt boys did not need to be told about menstruation, whilst boys expressed a big interest in learning and having more discussions about this topic, which was not the case in other studies [19, 47]. However, consistent with other reports, boys were rarely included in discussions about menstruation [19, 48]. This exclusion of boys can result in them having very negative views about menstruation as was found in Taiwan [48], which helps to perpetuate the stigma surrounding this element of reproductive health. Male members of the community also play a big role in the overall experience a woman has while menstruating, especially in patriarchal societies, where men are the main decision makers and control all the finances [19, 28]. However, there is limited data on their knowledge and attitudes towards menstruation. Engaging boys and other male members in strategies to improve MHM has the potential to make significant gains in addressing stigma and the reproductive health of their female counterparts.

The timing of learning about menstruation (pre versus post menarche) did not difference on feelings experienced when seeing menstrual blood for the first time. Most girls reported that they experienced a variety of negative feelings when they first saw menstrual blood. However, previous studies found prior knowledge at menarche was more likely linked to positive attitudes towards menstruation [1, 4, 15, 18, 49]. Girls in Mexico who had knowledge about menstruation prior to menarche were significantly more likely to know what was happening to their bodies and how to manage menses hygienically [27, 50]. One possible reason for this difference is that possibly girls in our study did not connect what they saw to what they had been taught or have heard about menstruation, suggesting girls the information that they received about menstruation may not be adequate or accurate. In addition, information alone in the absence of practical support may not be sufficient to address the challenges that girls face with social taboo surrounding menstruation, which can make girls starting menstruation to have negative feelings.

Menstruation is seen as a sign of fertility and maturity in these rural communities. As a result, for many girls, this may be a sign that they are ready for marriage and therefore have to leave school irrespective of whether or not they are willing or ready to do this [11, 51, 52]. These cultural beliefs can have negative impacts on a girl’s education and future prospects of economic and gender empowerment. Similar beliefs are also seen in other parts of Sub-Saharan Africa and Asia [1–4].

Girls in this study were more likely to use cloth rather than disposable pads. For some this was their preference, as was also seen in girls in India [9]. However, for most of them it was due to the lack of access to disposable pads for a number of reasons including: the pads were too costly, their school could not provide any, pads were unavailable in the village shops, they were too shy to purchase them from the village shops and did not know how to use the pads. Studies in Ethiopia [1] and India [53] found that girls could not use pads due to lack of awareness, mothers’ restrictions or disposal problems. In the Gambia, cultural views that the disposed blood-stained material could be used to curse you, could be an important reason to make women prefer the use of cloth over disposable pads. Taboos, secrecy and embarrassment associated with menstruation leads to difficulties in washing and drying menstrual absorbents hygienically. In most cases girls’ dry absorbent material in dark places or under the mattress to maintain secrecy. Such practices can increase the risk of infection or other health hazards. Concerns over poor washing and drying conditions were highlighted in previous studies as well [13, 20, 21, 23, 24]. This emphasises the need to break the silence surrounding menstruation, to allow girls to maintain adequate hygiene during this period.

Ideas of a girl being impure and unclean during menstruation resonated through all discussions. Some discussions suggested girls should not touch or interact with other because a girl is unclean during this period. They were also advised to avoid crowded places, therefore most girls tend to isolate themselves in their homes and potentially not attend school. This can then affect academic performance resulting in the girl dropping out of school [1]. Beliefs of impurity and restrictions associated with it have also been raised in previous studies [2, 21]. Addressing these beliefs can help in the attainment of SDG4 and SDG5 on ensuring inclusive and equitable education and gender equality and empowerment [41].

### Implications of the study to policy and practice

Although adolescents in this rural Gambian community had knowledge about menstruation, it was often inadequate and inaccurate. There is an urgent need to explore strategies to improve knowledge of menstruation among adolescents and adults in this community as well as address the stigma associated with it. Addressing problems girls face while menstruating therefore has the potential to promote gender equality and empowerment of women Sustainable Development Goal 5 (SDG 5) [54], with several knock on effects on most SDGs related to health (SDG3), education (SDG4), equality (SDG5 and SDG10), sanitation (SDG6), economic growth (SDG8) and responsible consumption (SDG12) [40, 41]. Our study had a number of limitations. We could only include one Arabic school in the study because most had closed by the time the study started therefore, we were not able to adequately explore the differences in MHM between adolescent girls in the Arabic and English schools. However, our findings provide some baseline information on potential difference that can be explored in greater detail in future studies to support more context specific MHM interventions. In addition, this study was conducted in the schools with access to health care services at the MRCG field station and may therefore not be representative views and experiences of adolescents in the wider Kiang West district of The Gambia. However, with the help of the regional education officer we tried to ensure that the schools selected were diverse and therefore represented the diversity of experiences of adolescents in the region.

Despite these limitations, this is the first study, to our knowledge, to collected in depth information about perceptions, practices and cultural beliefs about menstruation among communities in rural Gambia. The strengths of our work include: a good combination of qualitative and qualitative tools to explore and quantify some aspects of this complex topic, the use of exclusively same gender interviewers in the study to facilitate participating in the study and inclusion of different type of community members (boys, girls, mothers and teachers).

## Conclusion

The topic of menstruation in rural Gambia is surrounded by taboos, secrecy and embarrassment, which hinder adolescents from seeking advice from parents and teachers on appropriate MHM practices. Strategies to encourage positive social norms towards menstruation would help to promote more open discussions about it at the family, community and national level, which will contribute the creation of environments where girls can manage a healthy menstruation, with dignity, comfort and confidence.

## Additional files

Additional file 1: In depth interview guidelines (Guidelines developed by the team for mothers, teachers and boys). (DOCX 28 kb)

Additional file 2: Focus group discussion interview guidelines (Guidelines developed by the team for boys and girls). (DOCX 45 kb)

Additional file 3: Questionnaire (Questionnaire Tool used to collect the cross-sectional data among adolescent girls developed by the team in this study) (DOCX 199 kb)

## Abbreviations

DSS: Demographic Surveillance System
FGDs: Focus Group Discussions
IDI: In-depth Interviews
LMICs: Low- and middle-income countries
MHM: Menstrual hygiene management
MRCG: Medical Research Council The Gambia
SDGs: Sustainable Development Goals

## References

[1] Tegegne TK, Sisay MM. Menstrual hygiene management and school absenteeism among female adolescent students in Northeast Ethiopia. BMC Public Health [Internet]. 2014;14(1):1118. Available from: 10.1186/1471-2458-14-1118 10.1186/1471-2458-14-1118 PMC4232635 25355406

[2] Winkler IT, Roaf V. Taking the Bloody Linen out of the Closet – Menstrual Hygiene as a Priority for Achieving Gender Equality. Cardozo JL & Gender. 2014:1–54.

[3] Adinma ED, Adinma JI (2008). Perceptions and Practices on menstruation amongst Nigerian secondary school girls. Afr J Reprod Health.

[4] Mason L, Nyothach E, Alexander K, Odhiambo FO, Eleveld A, Vulule J, Rheingans R, Laserson KF, Mohammed A, Phillips-Howard PA. “We keep it secret so no one should know” - A qualitative study to explore young schoolgirls attitudes and experiences with menstruation in rural Western Kenya. PLoS One. 2013;8(11):e79132. 10.1371/journal.pone.0079132 PMC3828248 24244435

[5] Haver J, Caruso BA, Ellis A, Sahin M, Villasenor JM, Andes KL, Freeman MC. WASH in schools empowers girls’ education in Masbate Province and Metro Manila, Philippines: an assessment of menstrual hygiene management in schools. New York: UNICEF. 2013.

[6] Sumpter C, Torondel B. A Systematic Review of the Health and Social Effects of Menstrual Hygiene Management. PLoS One. 2013;8(4):e62004. 10.1371/journal.pone.0062004 PMC3637379 23637945

[7] Das P, Baker KK, Dutta A, Swain T, Sahoo S, Das BS, et al. Menstrual Hygiene Practices, WASH Access and the Risk of Urogenital Infection in Women from Odisha, India. Wilson BA, editor. PLoS One [Internet]. 2015 30 [cited 2018 Apr 3];10(6):e0130777. Available from: 10.1371/journal.pone.0130777 10.1371/journal.pone.0130777 PMC4488331 26125184

[8] Hulland KRS, Chase RP, Caruso BA, Swain R, Biswal B, Sahoo KC, et al. Sanitation, Stress, and Life Stage: A Systematic Data Collection Study among Women in Odisha, India. Kavushansky A, editor. PLoS One [Internet]. 2015 9 [cited 2018 Apr 3];10(11):e0141883. Available from: 10.1371/journal.pone.0141883 10.1371/journal.pone.0141883 PMC4638353 26551866

[9] Mathiyalagen P, Peramasamy B, Vasudevan K, Basu M, Cherian J, Sundar B. A descriptive cross-sectional study on menstrual hygiene and perceived reproductive morbidity among adolescent girls in a union territory, India. Journal of family medicine and primary care. 2017;6(2):360. 10.4103/2249-4863.220031 PMC5749087 29302548

[10] Phillips-howard PA, Caruso B, Torondel B, Sahin M, Sommer M, Caruso B (2016). Menstrual hygiene management among adolescent schoolgirls in low- and middle-income countries : research priorities schoolgirls in low- and middle-income countries. Glob Health Action.

[11] King E, Winthrop R. Today's challenges for girls' education. Brookings Global Working Paper Series. 2015.

[12] Republic of the gambia ministry of health and social welfare the gambia national strategy for sanitation and hygiene 2011–2016 [Internet]. 2011 [cited 2018 Dec 11]. Available from: http://washinschoolsmapping.com/wengine/wp-content/uploads/2015/10/Gambia-National-Strategy-for-Sanitation-and-Hygiene.pdf

[13] Crofts T, Fisher J. Menstrual hygiene in Ugandan schools: an investigation of low-cost sanitary pads. J Water, Sanit Hyg Dev [Internet]. 2012;2(1):50. Available from: 10.2166/washdev.2012.067

[14] Miiro G, Rutakumwa R, Nakiyingi-Miiro J, Nakuya K, Musoke S, Namakula J, Francis S, Torondel B, Gibson LJ, Ross DA, Weiss HA. Menstrual health and school absenteeism among adolescent girls in Uganda (MENISCUS): a feasibility study. BMC women's health. 2018;18(1):4. 10.1186/s12905-017-0502-z PMC5753466 29298699

[15] Cooper SC, Koch PB. “Nobody Told Me Nothin”: Communication About Menstruation Among Low-Income African American Women. Women Health [Internet]. 2007 Oct 11 [cited 2018 Apr 3];46(1):57–78. Available from: 10.1300/J013v46n01_05 10.1300/J013v46n01_05 18032175

[16] Lawan UM, Yusuf NW, Musa AB (2010). Menstruation and menstrual hygiene amongst adolescent school girls in Kano. Northwestern Nigeria Afr J Reprod Health.

[17] Houston AM, Abraham A, Huang Z, D’Angelo LJ (2006). Knowledge, attitudes, and consequences of menstrual health in urban Adolescent females. J Pediatr Adolesc Gynecol.

[18] Sommer M, Hirsch JS, Nathanson C, Parker RG (2015). Comfortably, safely, and without shame: defining menstrual hygiene management as a public health issue. Am J Public Health.

[19] Mahon T, Tripathy A, Singh N (2015). Putting the men into menstruation: The role of men and boys in community menstrual hygiene management. Waterlines..

[20] Management MH. Celebrating Womanhood : Highlights Report. 2013;(March).

[21] Thakur H, Aronsson A, Bansode S, Stalsby Lundborg C, Dalvie S, Faxelid E. Knowledge, practices, and restrictions related to menstruation among young women from low socioeconomic community in Mumbai, India. Frontiers in public health. 2014;2:72. 10.3389/fpubh.2014.00072 PMC4080761 25072044

[22] Boosey R, Prestwich G, Deave T (2014). Menstrual hygiene management amongst schoolgirls in the Rukungiri district of Uganda and the impact on their education: A cross-sectional study. Pan Afr Med J.

[23] Dasgupta A, Sarkar M. Menstrual hygiene: how hygienic is the adolescent girl?. Indian journal of community medicine: official publication of Indian Association of Preventive & Social Medicine. 2008;33(2):77. 10.4103/0970-0218.40872 PMC2784630 19967028

[24] Shah SP, Nair R, Shah PP, Modi DK, Desai SA, Desai L (2013). Improving quality of life with new menstrual hygiene practices among adolescent tribal girls in rural Gujarat. India Reprod Health Matters.

[25] Walraven G, Ekpo G, Coleman R, Scherf C, Morison L, Harlow SD. Menstrual Disorders in Rural Gambia. Stud Fam Plann [Internet]. 2002 1 [cited 2018 Dec 19];33(3):261–268. Available from: 10.1111/j.1728-4465.2002.00261.x 10.1111/j.1728-4465.2002.00261.x 12385087

[26] Gender Education Unit The Gambia. Study into the impact of the supply of sanitary pads on girls’ schooling. Gambia; 2011.

[27] Chandra-Mouli V, Patel SV. Mapping the knowledge and understanding of menarche, menstrual hygiene and menstrual health among adolescent girls in low-and middle-income countries. Reproductive health. 2017;14(1):30. 10.1186/s12978-017-0293-6 PMC5333382 28249610

[28] Mason L, Sivakami M, Thakur H, Kakade N, Beauman A, Alexander KT, van Eijke AM, Laserson KF, Thakkar MB, Phillips-Howard PA. ‘We do not know’: a qualitative study exploring boys perceptions of menstruation in India. Reproductive health. 2017;14(1):174. 10.1186/s12978-017-0435-x PMC5721687 29216895

[29] Demba E, Morison L, Van der Loeff MS, Awasana AA, Gooding E, Bailey R, Mayaud P, West B. Bacterial vaginosis, vaginal flora patterns and vaginal hygiene practices in patients presenting with vaginal discharge syndrome in The Gambia, West Africa. BMC infectious diseases. 2005;5(1):12. 10.1186/1471-2334-5-12 PMC1083415 15757510

[30] Hoare B. The Gambia. In: The Kingfisher A-Z Encyclopedia. Kingfisher Publications; 2002. p. 11.

[31] CIA U. Central Intelligence Agency-The World Factbook. New Zealand. 2016.

[32] THE WORLD BANK. Poverty Overview [Internet]. 2018 [cited 2018 Apr 17]. Available from: http://www.worldbank.org/en/topic/poverty/overview

[33] The World Bank. The Gambia Overview [Internet]. 2017 [cited 2018 Apr 3]. Available from: http://www.worldbank.org/en/country/gambia/overview

[34] MRC International Nutrition Group. West Kiang Demographic Surveillance System (DSS) [Internet]. [cited 2018 Apr 23]. Available from: http://ing.mrc.ac.uk/home/research-areas/west-kiang-demographic-surveillance-system-dss/

[35] Rayco‐Solon P, Moore SE, Fulford AJ, Prentice AM. Fifty‐year mortality trends in three rural African villages. Tropical medicine & international health. 2004;9(11):1151–60. 10.1111/j.1365-3156.2004.01325.x 15548310

[36] Kea P. Girl farm labour and double-shift schooling in the Gambia: the paradox of development intervention. Canadian Journal of African Studies/La Revue canadienne des études africaines. 2007;41(2):258–88.

[37] Saunders B, Sim J, Kingstone T, Baker S, Waterfield J, Bartlam B, Burroughs H, Jinks C. Saturation in qualitative research: exploring its conceptualization and operationalization. Quality & quantity. 2018;52(4):1893–907. 10.1007/s11135-017-0574-8 PMC5993836 29937585

[38] Elo S, Kyngäs H. The qualitative content analysis process. Journal of advanced nursing. 2008;62(1):107-15. 10.1111/j.1365-2648.2007.04569.x 18352969

[39] StataCorp. Texas, USA;

[40] United Nations Development Programme (UNDP). Sustainable Development Goals | UNDP [Internet]. 2016 [cited 2018 Dec 11]. Available from: http://www.undp.org/content/undp/en/home/sustainable-development-goals/

[41] SIMAVI P and WU. MHM and SDGs | MHDay - menstruation matters to everyone, everywhere [Internet]. 2018 [cited 2018 Dec 19]. Available from: http://menstrualhygieneday.org/project/infographic-mhm-and-sdgs/

[42] UNESCO. Good Policy and Practice in Health Education. Booklet 9 Puberty Education and Menstrual Hygiene Management. 2014 [cited 2018 Jul 2]; Available from: http://unesdoc.unesco.org/images/0022/002267/226792e.pdf

[43] McMahon SA, Winch PJ, Caruso BA, Obure AF, Ogutu EA, Ochari IA, Rheingans RD. 'The girl with her period is the one to hang her head' Reflections on menstrual management among schoolgirls in rural Kenya. BMC international health and human rights. 2011;11(1):7. 10.1186/1472-698X-11-7 PMC3129305 21679414

[44] Jewitt S, Ryley H (2014). It’s a girl thing: menstruation, school attendance, spatial mobility and wider gender inequalities in Kenya. Geoforum [Internet].

[45] Crichton J, Ibisomi L, Gyimah SO. Mother–daughter communication about sexual maturation, abstinence and unintended pregnancy: Experiences from an informal settlement in Nairobi, Kenya. J Adolesc. 2012;35(1):21–30. 10.1016/j.adolescence.2011.06.008 21783241

[46] Gultie T, Hailu D, Workineh Y. Age of menarche and knowledge about menstrual hygiene management among adolescent school girls in Amhara province, Ethiopia: implication to health care workers & school teachers. PLoS One. 2014;9(9):e108644. 10.1371/journal.pone.0108644 PMC4182550 25268708

[47] Pillitteri SP (2011). School menstrual Hygiene Management in Malawi.

[48] Chang YT, Hayter M, Lin ML. Pubescent male students’ attitudes towards menstruation in Taiwan: implications for reproductive health education and school nursing practice. Journal of Clinical Nursing. 2012;21(3–4):513–21. 10.1111/j.1365-2702.2011.03700.x 21457380

[49] Britton CJ (1996). Learning about “The curse”: an anthropological perspective on experiences of menstruation. Womens Stud Int Forum.

[50] Marván ML, Molina-Abolnik M. Mexican adolescents' experience of menarche and attitudes toward menstruation: role of communication between mothers and daughters. Journal of pediatric and adolescent gynecology. 2012;25(6):358–63. 10.1016/j.jpag.2012.05.003 22975203

[51] UNICEF Mozambique. Girls Not Brides Mozambique-Child Marriage and Adolescent Pregnancy in Mozambique [Internet]. 2015 [cited 2018 Apr 3]. Available from: https://www.girlsnotbrides.org/child-marriage/mozambique/

[52] Statistics of Girls’ Education - The Facts. Education for All Global Monitoring Report. 2013 [cited 2018 Apr 3]; Available from: https://en.unesco.org/gem-report/sites/gem-report/files/girls-factsheet-en.pdf

[53] Thakre SB, Thakre SS, Reddy M, Rathi N, Pathak K, Ughade S. Menstrual hygiene: knowledge and practice among adolescent school girls of Saoner, Nagpur district. J Clin Diagn Res. 2011;5(5):1027–33.

[54] Osborn D, Cutter A, Ullah F. Universal sustainable development goals. Understanding the Transformational Challenge for Developed Countries. 2015.

