# Supplementary material for: A rite of passage: a mixed methodology study about knowledge, perceptions and practices of menstrual hygiene management in rural Gambia
Source: BMC Public Health. 2019 Mar 7;19:277. doi: 10.1186/s12889-019-6599-2 (PMC6407285; doi:10.1186/s12889-019-6599-2)
Supplement: Supplementary file 1 — In depth interview guidelines (Guidelines developed by the team for mothers, teachers and boys). (DOCX 28 kb) [file 12889_2019_6599_MOESM1_ESM.docx]

**IDI Guide**

*Before turning on the recorder:

- Introduce yourself
- Go over the information leaflet and consent form
- Go over broadly what the interview will be about
- Assure them that there is no wrong answer, everything they say will be helpful – we are interested in knowing their views and it is in no way testing or judging them
- Ensure them that everything they say will be strictly confidential and will only be shared with people involved in the study.
- The data collected will be anonymised so others will not be able to identify them.
- Explain you may note things down as you go along, so that you can revisit these later

Informal conversation (intended to build rapport and trust between you and the participant):

- Talk to them as you would talk to a friend until you feel they are comfortable eg, ask how their day was, what the best part of the day was, do they enjoy going to school, what football team they support, what sports they play….etc

During the interview

- Once you start the interview; if feel the participant is holding back for some questions, remind them again about confidentiality of answers and that there is no wrong answer.
- If the participant doesn’t answer the question, give them some time to collect their thoughts and respond, but if you still do not get a response, check that they understand what you mean, ask them to repeat the question back to you to verify.
- If the participant says something interesting and relevant to the topic, ask them more about it, dig deeper into the area (even though it is not on the interview guide).

| **Interviews with teachers** | |
| --- | --- |
| Participant Number: _ _ _ | Date: |
| Gender: Male Female | Location: |
| 1. How long have you been working at this school? | |
| 1. Do you like to work at this school? | |
| 1. Do you talk about reproductive health at school? | |
| 1. Do you talk about menstruation-related issues at school (if No- *why?* skip to Q14) | |
| 1. If yes, how is menstruation-related topics discussed at the school?  - *As a component of the core curriculum (e.g., in science class)* - *As an integral part of a special module on healthy living/ life skills or sex education* - *As a stand-alone special module on menstrual hygiene exclusively* - *Through a school-sponsored extracurricular program (e.g., health clubs)* - *Only sporadically/informally/occasionally when girls ask* - *Only sporadically/informally/occasionally when teachers perceive girls need information* | |
| 1. If yes, who teaches menstrual-related education at the school? | |
| 1. If yes, can you explain what is covered in the education session?   Probe:   - *Information provided is about biological reasons of menstruation* - *Information provided teaches girls about managing menstruation, hygiene, how to clean/use cloths, etc.* - *Information provided includes information about reproductive health* | |
| 1. Is it taught to girls?  - If no, why not? - *Are there any information leaflets available-if so what kind?* | |
| 1. If yes, when and how often do girls receive menstrual-related education? | |
| 1. If yes, what grade do girls start receiving this information? (what age are the girls when the receive this information) | |
| 1. Are boys taught about reproductive and sexual health?   *Probe:*  *Is it taught in separate lectures or at the same time?* | |
| 1. When and how often do boys receive this information? | |
| 1. What information is covered?  - *Changes that occur during puberty* - *Safe sex* - *Menstrual issues* | |
| 1. Are teachers at this school trained on menstrual health and hygiene promotion/education? If yes, what kind of training do they receive? | |
| 1. Are menstruation-related education materials available and accessible to girls at the school?  - *What kind of material is available and where is it located?* | |
| 1. How do you think most teachers feel about teaching menstruation to girls in school?   If not taught, ask how they think teachers would feel if asked to teach about menstruation related topics   - *Would there be a difference in male teacher’s feelings vs. female teacher’s feelings?* | |
| 1. Do you know what material most girls at this school use to absorb menstrual blood? | |
| 1. Are sanitary napkins (or the most common absorbent used in the community) available for girls at the school in an emergency/accident situation?   *If yes:*   - *Who funds?* - *Do girls have to pay for them?* - *How are they distributed?* - *Do all the girls know this service is available?* | |
| 1. In many schools, girls get teased if it is known they are menstruating. Can you tell me the extent to which this happens at this school?   *Probe:*   - *Who teases?* - *Why is there teasing?* - *Are those caught teasing ever disciplines?* | |
| 1. Can you tell me how girls’ behaviours may change when they are menstruating?   *Probe:*   - *Class/activity participation* - *Missing school* - *Leaving school* - *Distraction* - *Class behaviour, etc* | |
| 1. What do you think are some of the reasons that girls miss school? | |
| 1. In many schools, girls miss school or leave school early when they are menstruating. How often does that happen at this school? | |
| 1. What do you think schools can do to make it easier for girls to be in school during menstruation?   *Probe:*   - *Education* - *Facilities (gender specific latrines, water, availability, privacy, absorbent disposal place/device)* - *Support* - *Resources, etc* | |
| 1. Have any girls approached you asking for advice on menstruation? If yes, what kind of questions have you been asked? | |
| 1. What do you think is the best way for girls to get information about menstruation and menstrual hygiene management? | |
| Thank you for your participation. Your involvement is really important to us. Before we end, do you have anything else you would like to share? | |

| **In-depth interview mothers** | |
| --- | --- |
| Participant Number: _ _ _ | Date: |
| **Personal background**  *(opening questions; intended to build rapport and gradually lead into the key questions)* | |
| **It would be great to know more about you** | |
| 1. Can you tell me your name and your age? | |
| 1. What year of education did you complete?  - *How old were you when you left school?* | |
| 1. How many children do you have? | |
| 1. How old are your children? | |
| 1. What is your favorite part of the day? | |
| 1. What is your favorite meal? | |
| **Key questions** | |
| 1. What do you know about menstruation?  - *What happens during menstruation?* - *Why does it happen?* - *What triggers it?* - *What should women do while they are menstruating?* | |
| 1. Do you think it is easy for women in the community to continue with their normal life when they are menstruating?  - *What would affect ability to continue normal life? --- water, privacy, pads, sanitation, comfort, social and religious restrictions)* - *Is there anything they cannot do while menstruating?* | |
| 1. What materials do you use to absorb menstrual blood?  - *Where do you get these materials from?* - *Why do you use them?* - *Do you like using them?* - *What do you do with the absorbents when you change them?* | |
| 1. What are your views on menstruation?  - *Should it be kept a secret from other women or men?* | |
| 1. What does menstruation signify in your community? | |
| 1. Do you talk to your daughters about menstruation?  - *Why?* - *What do you tell them?* - *When do you tell them (before or after menarche)?* | |
| 1. Do you feel it is easy to talk to girls about this topic? | |
| 1. Do you talk to your sons about menstruation?  - *Why?* - *What do you tell them?* | |
| 1. Do you think it is important for parents to talk to their children about menstruation? | |
| 1. What do your daughters or girls your daughters age know about menstruation? | |
| 1. What do you think is the best way for adolescents to learn about menstruation  - *In the school from teachers, from their friends, from their mothers, fathers or from other family members or from the mosque?* - *Do you think the same applies for boys and girls?* - *Is it important for boys to learn about menstruation?* | |
| 1. When do you think is the best time to talk to girls about menstruation? | |
| 1. What do you think can help improve the experience girls have when they start menstruating? | |
| 1. What do you feel are the main reasons girls are absent from school? | |
| 1. What do you feel are the main reasons girls drop-out of school? | |
| 1. Do you think it is important that girls go to school? Why? | |
| Thank you for your participation. Your involvement is really important to us, and we appreciate you sharing your personal experiences. Before we end, do you have any questions for me about menstruation, the interview, or other information we discussed? | |

| ***Start recorder*** |
| --- |
| **In-depth interview for boys** |
| **Personal background** |
| 1. Can you tell me your age and what grade are you in? |
| **Knowledge and attitudes of boys** |
| 1. Would you share with me the changes that have taken place in you both in your body and your thinking as you have grown up to become young adults? |
| 1. How about in girls, what changes you have noticed in them as they have grown up? |
| 1. Have you been able to share these changes with anyone else before?  - *Who did you talk to?* - *How did the conversation start?* - *What did you say/discuss?* - *Was it easy to talk to them about it?* - *Did you find the discussion helpful?* - *Who are you more likely to speak to about these changes, and why?* |
| 1. Do you know what menstruation is?  - *How do boys learn about menstruation?* - *Can you share with me what you were told and how you felt at the time?* - *What happens during menstruation?* - *Why does a girl menstruate?* - *What changes do you notice in a girl’s behavior when she menstruates?* - *When did you first hear about menstruation?* |
| 1. Do you feel a boy’s behavior towards a girl changes once she starts menstruating?  - *Why and How?* |
| 1. How do you feel when you hear others talking about menstruation?  - *Is there a difference between hearing boys talk about it and when you hear girls talking about it?* |
| 1. Would you share your views on menstruation?  - *Is there anything a girl cannot or should not do while menstruating?* - *Do you know why she has these restrictions?* - *How are boys expected to act around girls who are menstruating? And why?* |
| **Knowledge and attitudes of girls** |
| 1. Do you think girls in this area have enough information about menstruation?  - *How do you think the girls learn about it?* - *Do you think the girls need to be told more about it? Why?* - *Do you think they feel uncomfortable being at school when menstruating? Or do you think they may feel uncomfortable doing any activities while in school?* - *What do you think could be the reason for that?* - *Do you think girls miss school because of their period? Why do you think so?* |
| 1. Do you know if girls go to school when they are menstruating?  - *If they go, do they follow the normal routinely school activities?* - *Do you think there is any activity that they would feel uncomfortable doing while in school?* - *If they do not go, do you know why they do not go to school while menstruating?* |
| 1. Do you think they feel uncomfortable being at school when menstruating?  - *What do you think could be the reason for that?* |
| **Knowledge and attitudes of adults and recommendations** |
| 1. Do you feel that the adults may feel uncomfortable discussing these topics with the youth?  - *Why do you think this is so?* - *In your opinion, what do you think could help improve communication about such topics?* |
| 1. Do you think it is important for boys to know about menstruation?  - *Why?* |
| 1. Would you like to know more about menstruation?  - *Why?* |
| 1. What else would you like to know about menstruation? |
| 1. Who would you like to learn about it from?  - *Teachers; Parents; Sisters/Brothers; Other students* - *Is there any particular reason you would like to learn about it from this source?* |
| Thank you for your participation. Your involvement is really important to us, and we appreciate you sharing your views. Before we end, do you have any questions for me about menstruation, the interview, or other information we discussed? |
