# Supplementary material for: A rite of passage: a mixed methodology study about knowledge, perceptions and practices of menstrual hygiene management in rural Gambia
Source: BMC Public Health. 2019 Mar 7;19:277. doi: 10.1186/s12889-019-6599-2 (PMC6407285; doi:10.1186/s12889-019-6599-2)
Supplement: Supplementary file 2 — Focus group discussion interview guidelines (Guidelines developed by the team for boys and girls). (DOCX 45 kb) [file 12889_2019_6599_MOESM2_ESM.docx]

FGD Guide

*Before turning on the recorder:

- Introduce yourself
- Go over the information leaflet and consent form
- Go over broadly what the interview will be about
- Assure them that there is no wrong answer, everything they say will be helpful – we are interested in knowing their views and it is in no way testing or judging them
- Ensure them that everything they say will be strictly confidential and will only be shared with people involved in the study.
- The data collected will be anonymised so others will not be able to identify them.
- Explain you may note things down as you go along, so that you can revisit these later

Informal conversation (intended to build rapport and trust between you and the participant):

- Talk to them as you would talk to a friend until you feel they are comfortable eg, ask how their day was, what the best part of the day was, do they enjoy going to school, what football team they support, what sports they play….etc

During the interview

- Once you start the interview; if feel the participant is holding back for some questions, remind them again about confidentiality of answers and that there is no wrong answer.
- If the participant doesn’t answer the question, give them some time to collect their thoughts and respond, but if you still do not get a response, check that they understand what you mean, ask them to repeat the question back to you to verify.
- If the participant says something interesting and relevant to the topic, ask them more about it, dig deeper into the area (even though it is not on the guide).

| **Focus Group Discussion Guide (pre-menarche girls)** |
| --- |
| **Ice breaker** |
| **1. First, we would like to start with introductions. Can everyone introduce herself and tell us:**   - *What grade you are in?* - *Your favourite subject in school* - *Favourite colour* - *Favourite food* - *Favourite…. (continue till the girls start responding more openly)*   **2. What do you enjoy the most about school?** |
| **Key Questions** |
| **3. Can you all describe a typical school day for students, starting from when they arrive at school?**  *Probe*   - *What classes do students take?* - *When do you interact most with peers? Teachers?* - *How long is your school day? Are there breaks?* - *Do students ever go home during the school day?*   **4. Can you all describe the usual condition of the toilets/ facilities at this school?**  Probe:   - How many are there? - Are there separate toilets for boys and girls? - Comments on: cleanliness? Smell? Privacy? Safety? - Soap/water/materials available for personal hygiene? - Do girls prefer going with friends?   **5. Can you all explain any school rules regarding toilet use?**  Probe:   - Do all students use the toilets? - Are places besides the toilet used? Where? Why? - When can students use toilets? Breaks? During class? - Are toilets ever kept locked? Accessible? - If hauling water is needed, who does this? - Who cleans the toilets?   **6. What do girls in this community call menstruation?**   - *Are ‘secret’ words used so nobody else will know what is discussed?* - *What do these terms mean? Good or bad meanings?*   **7. How do girls in this community feel when they hear these words?**  **8. When do most girls learn about menstruation? From whom?**  **9. Is this a topic you can easily talk to other women at home or in school about?** *(grandmother, mother, teacher, sister, father…)*  **10. What do you know about menstruation?**  *Probe*   - Are there any stories told by grandmothers?   **11. What is menstruation?**   - Where does menstrual blood come from? - How often do women menstruate? - How long does a period last?   **12. What do girls know about the menstrual cycle?**  **13. Why do women menstruate?**   - Why don’t boys? - Do women menstruate when they are pregnant?   **14. What should a girl do when she starts bleeding?**  *Probe:*   - What should girls use to absorb blood? - How often should a girl change the material used to absorb blood? - Where is this material attached? - How is the material cleaned/disposed? - How can a girl keep clean during that period?   **15. Is there anything girls cannot do once they start menstruating?**   - Are there any restrictions to daily routine? If so, what? (household work, eat certain food, go out…..) - Are there any places she cannot go? Where? - Are there any places she cannot go on the school grounds?   **16. Can girls use the same toilet facilities while they are menstruating as what they normally use?**  **17. What is the source of this knowledge?** *(Mother, Teacher, Sister, Friends, Books..…)*  **18. What are your views on menstruation? Why?**  *Probe:*   - *Is it something to avoid or look down upon.*   **Thank you. We just have a few more questions.**  Closing Questions  **19. What are some of the worries girls have about having their first menstrual period?**  *Probe***:**   - Boys bullying - Shame - Fear - Guilt - May mean they have to stop studying and get married   **20. What additional information would girls like to have about menstruation?**  **21. Are girls excited to reach this phase of their woman-hood?**  *Probe:*   - - *Is it a natural wonderful part of being a woman (Why?)*   - *Or a curse (Why?)*     **22. Is there anything else that you would like to share or are there any questions you have for me?**  **~THANK YOU FOR YOUR TIME AND YOUR HELP~**  **NOTE: Keep the recorder on until all women leave your company. Sometimes they will stay and ask questions or give final thoughts that are particularly insightful.** |

| **Focus Group Discussion Guide (post-menarche girls)** |
| --- |
| **Ice breaker** |
| **1. First, we would like to start with introductions. Can everyone introduce herself and tell us:**   - *What grade you are in?* - *Your favourite subject in school* - *Favourite colour* - *Favourite food* - *Favourite…. (continue till the girls start responding more openly)*   **2. What do you enjoy the most about school?** |
| **Key Questions** |
| **3. Can you all describe a typical school day for students, starting from when they arrive at school?**  *Probe:*   - *What classes do students take?* - *When do you interact most with peers? Teachers?* - *How long is your school day? Are there breaks?* - *Do students ever go home during the school day?*   **4. Can you all describe the usual condition of the toilet facilities at this school?**  Probe:   - How many are there? - Are there separate toilets for boys and girls? - Comments on: cleanliness? Smell? Privacy? Safety? - Soap/water/materials available for personal hygiene? - Do girls prefer going with friends? - Can girls dispose of personal supplies? How?   **5. Can you all explain any school rules regarding toilet use?**  Probe:   - Do all students use the toilets? - Are places besides the toilet used? Where? Why? - When can students use toilets? Breaks? During class? - Are toilets ever kept locked? Accessible? (are there any times a girl cannot use the toilets) - If hauling water is needed, who does this? - Who cleans the toilets? |
| **Thank you. I would now like to discuss menstruation practices.**  **6a. What do girls in this community call menstruation?**   - *Are ‘secret’ words used so nobody else will know what is discussed?* - *What do these terms mean? Good or bad meanings?*   **6b. How do girls in the community perceive menstrual blood?**  **7. How do girls in this community feel when they hear these words?**  **8. When do most girls learn about menstruation? From whom?**  **9. What do girls know about menstruating before they start?**  *Probe:*   - Are there any stories told by grandmothers? - Why do women menstruate? - Do women menstruate when they are pregnant? - What is menstrual blood and where does it come from? - Is there anything women are not allowed to do while they menstruate   **10a. Do girls feel prepared or ready when they reach menarche?**  ***10b. How do most girls react when seeing menstrual blood for the first time?***  **11. Please explain to me what girls in this community generally do if they notice they have started menstruating?**  *Probe:*   - - *What is the first thing a girl will do?*   - *When will she do this? (Right away, at a more convenient time…)*   **12. What will she use to absorb the blood?**  *Probe:*   - *Where do girls get this material?*   - *What do girls use to hold the material in place? (Underwear? Used all the time?)*   - *Where will she go to put the material on?*   - *Would this be the same whether they started menstruating in school or at home?*   **13. What do girls think about buying materials for menstruation?**  *Probe:*   - - What is the cost? *(try establish if there are non-monetary costs)*   - What do women think about the cost?   - How accessible are they?   **14. Please tell me about any activities that may be changed while girls are menstruating?**   - - *Can you explain if restrictions interfere with school tasks?*   - *How do women feel about the restrictions?*   - *Does it make girls miss some days of school?*   - *Can you explain if girls feel it changes their performance in school? Why?*   **15. Can you explain to me what a girl in this community would do if she needs to change her absorbent materials?**  *Probe:*   - *How often does a girl change the material used?* - *Where do women change materials? (Probe to see if there are many)* - *Why do women change in that place?* - *How often would she change it on the more heavy bleeding days?*   **16. If a girl is going to reuse the material, what does she do with the** **used materials**?  *Probe:*   - *Where are materials washed?* - *When are materials washed?* - *Where are materials dried?*   **17. How is the reusable absorbents stored?**  *Probe:*   - *Where are materials stored?* - *Are materials wrapped in anything when stored?* - *Do they wash cloth with other clothes?*   **18. If a girl is NOT going to reuse her materials, where will she dispose of them?**   - *In toilet? Burned? Rubbish Pile? River/ pond? Countryside?* - *Why is it disposed here?*   **19. If women in this community are menstruating, where do they go to urinate and defecate?**  *Probe:*   - *Will they go to the same place as when they are not menstruating?* - *Do women try to go less frequently? Why?* - *Do women go at specific times? Why?*     **20. How do women change any of their practices in school while menstruating? Why?**  **21. How are practices of *young girls* different than those of women during menstruation?**   - What materials do they use? - Where do they change? - Where do they urinate? Defecate? - Are these places different than when they are not menstruating?   Aim is to understand what girls would like for an ideal toilet at school. Girls are given materials to independently or collaboratively draw their ideal toilet  **Activity**  End of activity  We would like to learn more about the toilet/bathroom you would like to have in school, particularly the characteristics that would be useful when menstruating.  Please draw your ideal toilet. Please thing about:   - characteristics of each stall as well as the whole block, - location - distance from classroom - use labels if you would like to   Once complete talk through the drawings with the girls  Ask about the following characteristics if they have not been mentioned:   - Privacy - Disposal - Light - Safety - Cleanliness - Location - Water inside - Place to wash materials or hands inside/nearby - Shower inside or nearby   Conclude with the following questions:   - Does your school have any toilets that look like this? - Do any of these features exist? - What do you school toilets need that they don’t have? What is the most important?   *End of activity*  **22. What are some of the reasons girls miss school?**  *Probe:*   - Lack of money - Have to stay home to help with household work - Early marriage - Lack of facilities to maintain adequate menstrual hygiene - Pain (during menstruation)   **23. How do you think girl’s school attendance will change if they had a separate private toilets? Why?**  **24. What do people in this community think about using toilets during menstruation?**  *Probe:*   - What do women think? - What do men think about women using the toilet during menstruation? - What do people think about urinating in the toilet during menstruation? - What do people think about defecating in the toilet during menstruation? |
| **Closing Questions** |
| **Thank you. We just have a few more questions.**  **25. What are some of the worries that girls have about going to school during their period?**  **26. What other changes do girls think would help improve school attendance?**  **27. How do you think women in this community would feel about answering questions in a survey about menstruation?**   - Do you think it is difficult for women to give honest answers? - What might help women answer these questions? - Would it make a difference if the person asking questions is male or female?   **28. Is there anything else that you would like to share or are there any questions you have for me?**  **~THANK YOU FOR YOUR TIME AND YOUR HELP~**  **NOTE: Keep the recorder on until all women leave your company. Sometimes they will stay and ask questions or give final thoughts that are particularly insightful.** |

| **Focus Group Discussion Guide (boys)** |
| --- |
| **Ice breaker** |
| 1. **First, we would like to start with introductions. Can everyone introduce themselves and tell us:**  - *What grade you are in?* - *Your favourite subject in school* - *Favourite colour* - *Favourite food* - *Favourite…. (continue till the boys start responding more openly)*  1. **What do you enjoy the most about school?** |
| **Key Questions** |
| **Thank you all for sharing. I would now like to talk about the differences in puberty between boys and girls.**   1. **Do you think there are any changes that occur to boys as they become teenagers/young adults? If so what?** 2. **What about girls, are there any changes that occur as they become teenagers/young adults?**   *Probe:*   - *Changing body shape* - *Menstruation*  1. **Do boys talk about these changes with each other** 2. **Do boys talk about these changes with their mother, father, sister or school teachers or friends?** 3. **What do you call monthly bleeding that girls go through?** 4. **What comes to mind when you hear that word? (ref menstruation)** 5. **How do boys in this community feel when they hear that word (menstruation)?** 6. **Are there any terms boys use to refer to menstruation? What are they?**   *Probe:*   - *What do these terms mean/symbolise, good or bad meaning?* - *Who uses those terms in your community?* - *Where did you learn these terms? From whom?*  1. **When do most boys first hear about menstruation?** 2. **Where do boys learn about menstruation? From whom?**   *Probe:*   - *Do parents or teachers talk about menstruation, with their sons?*  1. **Do boys know when a girl is menstruating? How do boys know?**   *Probe:*   - *How does she act?* - *Does a girl at school act differently during her period?* - *Why do you think she acts differently?* - *Does she act differently with boys? How so* - *Does she act differently with her girl-friends? How so?* - *Do girls talk to boys about menstruation?*  1. **How is menstruation talked about at school by boys?**   *Probe:*   - *What do boys say?* - *What do male teachers say? Female teachers?* - *Is it important for boys to understand menstruation?* - *Do boys freely talk about menstruation?* - *Is it acceptable for boys to talk about menstruation?*  1. **Do boys know why women menstruate?**  - *How often do they menstruate?*  1. **How are girls expected to act once they start menstruating?**   *Probe:*   - *Are there restrictions to daily routine? If so, what?* - *Are there places she cannot go? Where?* - *Are there places she cannot go on school grounds?*  1. **What are your views on menstruation? Why?**   *Probe:*   - *Is it something to avoid or look down upon.*  1. **How do boy’s behaviour towards girls change after she starts menstruating? Why?**   *Probe:*   - *What if a boy’s sister, cousin or friend started her menstruation, how would he treat her? Would he change his behaviour? How? Why?*  1. **How are boys expected to act around menstruating girls?**   *Probe:*   - *How did you learn this behaviour?* - *From observation? teachers? family? peers?* - *Does this behaviour change as boys / girls get older?*  1. **Sometimes boys tease girls when they have their period, can you tell me about a time that may have happened at this school**   **Probe:**   - *What do they say? Why did they do it?* - *Can you tell me a story of a girl who was once teased; don’t mention her name?* - *How do teachers react if they see boys teasing?*   **Thank you. We just have a few more questions.**  **Closing Questions**   1. **Do boys wish they are taught more about puberty and the changes that come with it** 2. **What would boys like to know?** 3. **Is there anything else that you would like to share or are there any questions you have for me?**   **~THANK YOU FOR YOUR TIME AND YOUR HELP~**  **NOTE: Keep the recorder on until all women leave your company. Sometimes they will stay and ask questions or give final thoughts that are particularly insightful.** |
|  |
