# Supplementary material for: A rite of passage: a mixed methodology study about knowledge, perceptions and practices of menstrual hygiene management in rural Gambia
Source: BMC Public Health. 2019 Mar 7;19:277. doi: 10.1186/s12889-019-6599-2 (PMC6407285; doi:10.1186/s12889-019-6599-2)
Supplement: Supplementary file 3 — Questionnaire (Questionnaire Tool used to collect the cross-sectional data among adolescent girls developed by the team in this study) (DOCX 199 kb) [file 12889_2019_6599_MOESM3_ESM.docx]

| **Questionnaire for girls** | | | | | | |
| --- | --- | --- | --- | --- | --- | --- |
| Participant Number : _ _ _ _ _ _ _ _ _ | | | Date : _ _ / _ _ / _ _ | | | |
| Location: | | | Age (years): _ _ | | | |
| **General Information** | | | | | | |
| 1. Standard Year of education: _ _ _ _ _ _ _ _ _ | | | | | | |
| 1. Religion: Muslim Christian Other _ _ _ _ _ _ _ _ _ _ | | | | | | |
| 1. How long does it take to reach school? _ _ _ _ _ _ _ _ _ _ _ | | | | | | |
| 1. Have you started your period?   Yes No (skip to Q6) | | | | | | |
| 1. In what year did you have your first period? _ _ _ _ | | | | | | |
| 1. What is your main water source at home? *Ilee jiwoo bumbaa muŋ minee ti suwokono?*   Household standpipe Household borehole Community standpipe  Community borehole Protected well Rain water  River water Unprotected well Drum | | | | | | |
| 1. What is the main toilet facilities members of your household usually use?   Flush toilet Pit latrine with a slab  Pit latrine without a slab Communal latrine  Bucket Use the bush | | | | | | |
| 1. What is the standard year of education that your **mother** has completed?   No formal education Some secondary (7-10th year)  Some primary (1-4th year) Completed secondary (13th year)  Completed primary (6th year) Further education (university, etc)  Arabic I don’t know | | | | | | |
| 1. What is the standard year of education that your **father** has completed?   No formal education Some secondary (7-10th year)  Some primary (1-4th year) Completed secondary (13th year)  Completed primary (6th year) Further education (university, etc)  Arabic I don’t know | | | | | | |
| **Knowledge and attitudes** | | | | | | |
| 1. What grade/year did you learn about menstruation? _ _ _ _ | | | | | | |
| 1. Who did you learn about menstruation from? (select all appropriate answers)   Grandmother Mother Sister Father Teacher  Other _ _ _ _ _ _ _ _ _ _ _ _ _ _ _ _ _ _ _ _ _ _ _ _ _ _ _ _ _ _ _ _ _ _ _ _ _ _ _ | | | | | | |
|  | Yes No I don’t know | | | | | |
| 1. Women stop menstruating as they grow very old? |  | | | | | |
| 1. Menstruation is a disease |  | | | | | |
| 1. Pregnant women menstruate |  | | | | | |
| 1. Menstrual blood comes from the stomach where the food is digested |  | | | | | |
| 1. Menstrual blood comes from the womb |  | | | | | |
| 1. Menstrual blood contains dangerous substances |  | | | | | |
| 1. Pain during menstruation means that someone is sick |  | | | | | |
| 1. It is harmful for a woman’s body if she runs or dances during her period |  | | | | | |
| 1. What do you think about menstruation?   It is a natural part of being a woman It is a curse  Other _ _ _ _ _ _ _ _ _ _ _ _ _ _ _ _ _ _ _ _ _ _ _ _ _ _ _ _ _ _ _ _ _ _ _ _ _ | | | | | | |
| 1. Are you/were you afraid to start menstruating? | Yes No | | | | | |
| 1. What are/were your fears of starting menstruating? (tick all the appropriate ones)   I felt I didn’t know enough to manage it well  Bullying/teasing  Shame  Fear of staining  Guilt  Sign that you will have to stop studying and get married  Pain  Other _ _ _ _ _ _ _ _ _ _ _ _ _ _ _ __ _ _ _ _ _ _ _ _ _ _ _ _ _ _ _ _ _ _ _ _ _ _ _ _ _ _ _ _ _ _ _ _ _ _ _ _ _ _ _ _ _ _ _ _ _ _ _ _ _ _ _ _ _ _ _ _ _ _ _ _ | | | | | | |
| **Missing school - It can often be difficult to get to school every day, even when you are trying your hardest** | | | | | | |
| 1. How many days of school have you missed in the last 2 months?   0 ½ 1 2 3 4 5 6 7 8 9 10 10+ | | | | | | |
| 1. In a normal month how many days do you miss of school because of illness?   0 ½ 1 2 3 4 5 6 7 8 9 10 10+ | | | | | | |
| 1. In a normal month how many days do you miss of school because of lack of money?   0 ½ 1 2 3 4 5 6 7 8 9 10 10+ | | | | | | |
| 1. In a normal month how many days do you miss of school because of burden of domestic duties?   0 ½ 1 2 3 4 5 6 7 8 9 10 10+ | | | | | | |
| 1. In a normal month how many days do you miss of school because of your period?   N/A – pre-menarche girl  0 ½ 1 2 3 4 5 6 7 8 9 10 10+ | | | | | | |
| 1. Are there any other reasons you personally miss school?   _ _ _ _ _ _ _ _ _ _ _ _ _ _ _ _ _ _ _ _ _ _ _ _ _ _ _ _ _ _ _ _ _ _ _ _ _ _ _ _ _ _ _ _ _ _ _ _ _ _ _ _ _ _ _ _ _ _ _ _ _ _ _ _ _ _ _ _ _ _ _ _ _ _ _ _ _ _ _ _ _ _ _ _ _ _ _ _ _ _ _ _ _ _ _ _ _ _ _ _ _ _ _ _ _ _ _ _ _ _ _ _ _ _ _ _ _ _ _ _ _ _ _ _ _ _ _ _ _ _ _ _ _ _ _ _ _ _ | | | | | | |
| **Absorbent material** | | | | | | |
| 1. Which of these products used to absorb menstrual blood have you heard of?   Disposable sanitary pad  Tampon  Menstrual cup  Reusable Pad (you can use again) made from old cloth or towel  Commercially available reusable Pad (you can use again) | | | | | | |
| 1. What do **your family members** normally use during your period?   Cloth/Towel Purchased sanitary pad Hand-made sanitary pads    Natural materials (mud, cotton or leaves)  *Alaŋdar feŋo (potoo, kotondoo waraŋ jamboo)*  Toilet paper  I don’t know  **End for pre-menarche girls** | | | | | | |
| 1. What do you normally use during your period?   Cloth/Towel Purchased sanitary pad Hand-made sanitary pads    Natural materials (mud, cotton or leaves)  *Alaŋdar feŋo (potoo, kotondoo waraŋ jamboo)*  Toilet paper | | | | | | |
| 1. Have you or a family member bought disposable sanitary pads from a shop in the last six months? | | | | | Yes No | |
| 1. Have you ever wanted to buy disposable sanitary pads from a shop but been unable to? | | Yes  No (skip to Q35) | | | | |
| 1. Why were you unable to buy disposal sanitary products from the shop?   I did not have enough money to buy disposable sanitary products from a shop.  There were no disposable sanitary products in the shops.  I felt embarrassed to go buy sanitary products from the shop  Other _ _ _ _ _ _ _ _ _ _ _ _ _ _ _ _ _ _ _ _ _ _ _ _ _ _ _ _ _ _ _ _ _ _ _ _ _ _ | | | | | | |
| 1. Does your school give girls a supply of pads? | | Yes  No (skip to Q37) | | | | |
| 1. How many pads are you provided with each month? _ _ _ | | | | | | |
| 1. How often would you change the absorbent material on one of the more heavy days of bleeding?   Twice a day  Once a day Three times a day or more  Other_ _ _ _ _ _ _ _ _ _ _ _ _ _ _ _ _ _ _ _ _ _ _ _ _ _ _ _ _ _ _ _ _ _ _ _ _ _ _ | | | | | | |
| 1. Do you feel you can change your absorbents in school? | | | | Yes No | | |
| 1. Where do you change your absorbent once you are at school?   Toilet Classroom Wait till you reach home  Other _ _ _ _ _ _ _ _ _ _ _ _ _ _ _ _ _ _ _ _ _ _ _ _ _ _ _ _ _ _ _ _ _ _ _ _ | | | | | | |
| 1. What do you do with absorbent material if you change it at school?   Keep it and bring it at home for future use  Wash it and use it again at school  Wash It and dry it at school  Dispose it (skip to Q45)  Other_ _ _ _ _ _ _ _ _ _ _ _ _ _ _ __ _ _ _ _ _ _ _ _ _ _ _ _ _ _ _ _ _ _ _ _ _ _ _ _ _ _ _ _ _ _ _ _ _ _ _ _ _ _ _ _ _ _ _ _ _ _ _ _ _ _ _ _ _ _ _ _ _ _ _ _ _ _ | | | | | | |
| 1. If the material is reused, how is it washed at school? (please select the main one)   With water   With water and soap/detergent  With water and mud/ash  Other_ _ _ _ _ _ _ _ _ _ _ _ _ _ _ __ _ _ _ _ _ _ _ _ _ _ _ _ _ _ _ _ _ _ _ _ | | | | | | |
| 1. Is it washed differently at home as compared to at school?   Yes … How _ _ _ _ _ _ _ _ _ _ _ _ _ _ _ _ _ _ _ _ _ _ _ _ _ _ _ _ _ _ _ _  No | | | | | | |
| 1. After washing it, how is it dried? (please select the main one)   Dry it in the sun or open space  Dry it inside the house or a room  I don’t dry it  Other _ _ _ _ _ _ _ _ _ _ _ _ _ _ _ __ _ _ _ _ _ _ _ _ _ _ _ _ _ _ _ _ _ _ _ _ _ _ | | | | | | |
| 1. Can the material be dried in school? | | | | Yes No | | |
| 1. Where do you dispose your sanitary pad (once you have finished using it?)   Put it in the pond  Inside a latrine  In a rubbish bin inside or close to the latrine  Discard in any open space  I burn it  In the household rubbish bin  *Other* | | | | | | |
| **Activities your period makes you miss (Skip for pre-menarche girls)** | | | | | | |
| 1. Does your period make you miss housework? | | Yes No | | | | |
| 1. Are there any other activities your period makes you miss? | | Yes  No (skip to Q49) | | | | |
| 1. What activities do you miss?   _ _ _ _ _ _ _ _ _ _ _ _ _ _ _ _ _ _ _ _ _ _ _ _ _ _ _ _ _ _ _ _ _ _ _ _ _ _ _ _ _ _ _ _ _ _ _ _ _ _ _ _ _ _ _ _ _ _ _ _ _ _ _ _ _ _ _ _ _ _ _ _ _ _ _ _ _ _ _ _ _ _ _ _ _ _ _ _ _ _ _ _ _ _ _ _ _ _ _ _ _ _ _ _ _ _ _ _ _ _ _ _ _ _ _ _ _ _ _ _ _ _ _ _ _ _ _ _ _ _ _ _ _ _ _ _ _ _ _ _ _ _ _ _ _ _ _ _ _ _ _ _ _ _ _ _ _ _ _ _ _ _ _ _ _ _ _ _ _ _ _ _ _ _ _ _ _ _ _ _ _ _ _ _ _ _ _ _ _ _ _ _ _ _ _ _ _ _ _ _ _ _ _ _ _ _ _ _ _ _ _ _ _ _ _ _ _ _ _ _ _ _ _ _ _ | | | | | | |
| 1. Average level of pain experienced during your period? *(10 being the worst pain you have ever experienced and 0 being pain free)*   0 ½ 1 2 3 4 5 6 7 8 9 10 | | | | | | |
| 1. You miss school during my period because: *(please select all that apply)*   I am afraid of staining my clothes  I am afraid of others making fun of me  I am afraid it makes me smell bad  Periods cause me a lot of pain  Periods can make me feel uncomfortable or tired.  There isn’t anywhere for girls to wash and change at school  There is nowhere to dispose of sanitary products.  I am afraid others will suspect I am on my period and will tease me | | | | | | |
| **School facilities (Skip for pre-menarche girls)** | | | | | | |
|  | | | | | | **Yes No** |
| 1. Is there soap and water available for personal hygiene? | | | | | |  |
| 1. Are the latrines ever kept locked? | | | | | |  |
| 1. Can girls dispose of menstrual absorbents in school? | | | | | |  |
| 1. Where can girls dispose menstrual absorbents when in school?   Pit latrine Old toilet Bin Take it home  Other _ _ _ _ _ _ _ _ _ _ _ _ _ _ _ _ _ _ _ _ _ _ _ _ _ _ _ _ _ _ _ _ _ _ _ _ _ | | | | | | |
| 1. Do you feel comfortable using the school latrines?   Yes always Not when I am menstruating Never | | | | | | |
| 1. Who cleans the latrines?   Students Teachers Parents Caretaker/cleaner  I don’t know Other _ _ _ _ _ _ _ _ _ _ _ _ _ _ _ _ _ _ _ _ _ _ _ _ _ _ _ | | | | | | |
| **Attitudes, self-esteem and feelings (Skip for pre-menarche girls)** | | | | | | |
| 1. How did you feel the first time you saw menstrual blood? (select all that are appropriate)   Scared Shocked Disgusted Ashamed  Other _ _ _ _ _ _ _ _ _ _ _ _ _ _ _ _ _ _ _ _ _ _ _ _ _ _ _ _ _ _ _ _ _ _ _ _ _ | | | | | | |
|  | | | | | | Yes No |
| 1. During my period I am happy with myself. | | | | | |  |
| 1. During my period I feel I am no good. | | | | | |  |
| 1. During my period I feel that I am a failure. | | | | | |  |
| 1. During my period I feel ashamed. | | | | | |  |
| 1. During my period I feel less confident than when I am not on my period. | | | | | |  |
